# CK2 Phosphorylating I_2_^PP2A^/SET Mediates Tau Pathology and Cognitive Impairment

**DOI:** 10.3389/fnmol.2018.00146

**Published:** 2018-04-30

**Authors:** Qing Zhang, Yiyuan Xia, Yongjun Wang, Yangping Shentu, Kuan Zeng, Yacoubou A. R. Mahaman, Fang Huang, Mengjuan Wu, Dan Ke, Qun Wang, Bin Zhang, Rong Liu, Jian-Zhi Wang, Keqiang Ye, Xiaochuan Wang

**Affiliations:** ^1^Key Laboratory of Education Ministry of China for Neurological Disorders, Department of Pathophysiology, School of Basic Medicine, Tongji Medical College, Huazhong University of Science and Technology, Wuhan, China; ^2^Department of Pathology and Laboratory Medicine, School of Medicine, Emory University, Atlanta, GA, United States; ^3^Co-innovation Center of Neuroregeneration, Nantong University, Nantong, China; ^4^Department of Genetics and Genomic Sciences, Icahn School of Medicine at Mount Sinai, New York, NY, United States

**Keywords:** casein kinase 2 (CK2), Alzheimer disease (AD), SET, tau phosphorylation, cognitive impairment

## Abstract

Casein kinase 2 (CK2) is highly activated in Alzheimer disease (AD) and is associated with neurofibrillary tangles formation. Phosphorylated SET, a potent PP2A inhibitor, mediates tau hyperphosphorylation in AD. However, whether CK2 phosphorylates SET and regulates tau pathological phosphorylation in AD remains unclear. Here, we show that CK2 phosphorylating SET at Ser9 induced tau hyperphosphorylation in AD. We found that either Aβ treatment or tau overexpression stimulated CK2 activation leading to SET Ser9 hyperphosphorylation in neurons and animal models, while inhibition of CK2 by TBB abolished this event. Overexpression of CK2 in mouse hippocampus via virus injection induced cognitive deficit associated with SET Ser9 hyperphosphorylation. Injection of SET Ser9 phosphorylation mimetic mutant induced tau pathology and behavior impairments. Conversely co-injection of non-phosphorylated SET S9A with CK2 abolished the CK2 overexpression-induced AD pathology and cognitive deficit. Together, our data demonstrate that CK2 phosphorylates SET at Ser9 leading to SET cytoplasmic translocation and inhibition of PP2A resulting in tau pathology and cognitive impairments.

## Introduction

Casein kinase 2 (CK2) is highly ubiquitous, evolutionarily conserved in eukaryotic cells (Mathias, 2010). The CK2 holoenzyme is a serine/threonine protein kinase composed of two catalytic (α/α′) and two regulatory (β) subunits (Litchfield, 2003). CK2 has been implicated in numerous aspects of neural function, including neuronal survival, regulation of some neurotransmitters’ receptors, circadian rhythm, and higher brain functions such as learning and memory (Blanquet, 2000). Overexpression of CK2 has been linked to several pathological conditions, ranging from cardiovascular pathologies (Kohlstedt et al., 2002) and cancer progression (Ahmad et al., 2005) to infectious diseases (Guerra and Issinger, 2008) and neurodegeneration (Perez et al., 2011). As one of the first protein kinases identified in Alzheimer disease (AD) accumulating evidence demonstrated that CK2 is associated with neurofibrillary tangles (NFTs) degeneration. CK2 immunoreactivity was shown to be significantly (22%) increased in NFTs-bearing hippocampal neurons with strong anti-tau immunolabeling in comparison with those without NFTs (Rosenberger et al., 2016). Compared to non-demented controls, CK2 level in the hippocampus and temporal cortex of AD patients is markedly increased (Rosenberger et al., 2016). At the ultrastructural level CK2 was immunolocalized to the paired helical filaments (PHFs) of the tangle-bearing neurons, as well as to PHF in neuropil threads and some dystrophic neurites in plaques (Masliah et al., 1992). These studies strongly suggest that CK2 hyper-activation may be involved in the pathology of AD.

Being a key protein phosphatase in dephosphorylating tau, protein phosphatase-2A (PP2A) is compromised in the AD brains (Liu et al., 2005; Liang et al., 2008), while the level of its endogenous specific inhibitor 2 (I_2_^PP2A^), also known as SET, is increased (Tanimukai et al., 2005). SET is widely expressed in different tissues and localized primarily in the nucleus (Tanimukai et al., 2004), where it mainly protects histones from acetylation by histone acetyl transferases (Canela et al., 2003), modulates HuR mRNA binding, regulates G2/M transition via binding to p21CIP1, and acts as a transcription factor for P450c17 activation (Cho et al., 2010). In the AD brains, SET is translocated from its primary nuclear location to the cytoplasm in the neurons and co-localizes with both PP2A and abnormally hyperphosphorylated tau which forms NFTs in the neuronal cytoplasm (Tanimukai et al., 2005; Tsujio et al., 2005; Polydoro et al., 2013). Our previous study also showed that SET is phosphorylated at Ser9 in AD brains and this phosphorylation induces its cytoplasmic detention, inhibition of PP2Ac subsequently leading to tau hyperphosphorylation in HEK293/tau cells (Yu et al., 2013). CK2 co-localizes with NFTs (Masliah et al., 1992) while SET mediates the formation of NFTs consisting of hyperphosphorylated tau (Arif et al., 2014), indicating that there might be a cross-talk between CK2 and SET in AD pathogenesis. In current study, we investigated the possibility that CK2 indeed phosphorylates SET in AD and mediates its inhibitory activity toward PP2A, resulting in tau pathology and cognitive impairments.

## Materials and Methods

### Plasmids, Viruses, Chemicals, and Antibodies

Site-directed mutations were constructed using the QuikChange site-directed mutagenesis kit (Stratagene) according to the manufacturer’s instructions. Site-directed mutagenesis was carried out to mutate Ser9 of SET to either alanine (S9A) or glutamic acid (S9E), which mimicked the non-phosphorylated or phosphorylated condition of SET respectively. All mutants were generated by PCR and cloned in a His-tagged pcDNA3.1 (-) vector respectively in Xho I and Kpn I restriction sites. We transfected cells with plasmid coding for CK2α, which corresponds to catalytic alpha subunit. All plasmids were sequenced and prepared using an endotoxin-free plasmid extraction kit (Tiangen). AAV2-hTau (full-length 441- amino acid human 4 repeat tau), AAV2-pCAG-SET WT, AAV2-pCAG-SET S9A, AAV2-pCAG-SET S9E, and AAV2-pCAG-CK2 were constructed and packaged by Obio Technology (Shanghai, China) Co., Ltd. Lipofectamine 2000 transfection reagents were from Invitrogen. Bicinchoninic acid (BCA) protein detection kit was from Pierce (Rockford, IL, United States). Reagents for cell culture were from Gibco BRL (Gaithersburg, MD, United States). Antibodies employed in this study are listed in **Supplementary Table S1**.

### Animals

3×Tg AD mice (PS1m146v/APPswe/TauP301L) were purchased from the Jackson Laboratory. APP/PS1 mice were from the Model Animal Research Center of Nanjing University. Male C57BL/6 mice (2-month old, 23 ± 2 g) were supplied by the Experimental Animal Central of Wuhan University. All the animals were housed in an air-conditioned room (22 ± 2°C, 12-h light/dark cycle) with free access to food and water. The behavioral tests were performed on their active hours.

### PP2A Activity Assay

PP2A activity in the cell extracts and brain lysates were measured using the phosphatase kit V2460 according to the manufacturer’s procedure (Promega).

### Fear Conditioning Trial

Fear conditioning was carried out as described previously (Kass et al., 2013). Mice were placed into a square chamber with a grid floor. On the first day (day 1), each mouse was habituated to the chamber for 3 min, and then given a foot shock (0.8 mA, 2 s). Then the mice were returned to their home cages. After 1 h, the mice were put into the same chamber without any stimulus for 3 min, freezing time during the 3 min was recorded for assessment of memory. The next day (day 2) the mice were exposed to the same chamber without foot shock for 3 min and freezing time was recorded.

### Morris Water Maze Test

The standard Morris water maze (MWM) procedure was used with minor modifications as described previously (Morris, 1984). The water maze test contains acquisition training and probe trial. During the acquisition training, the mice were trained to find a submerged platform hidden 1 cm under water by using constant cues outside the pool. During each trial, mice have up to 60 s to find the hidden platform; otherwise, it would be guided to the platform and forced to stay on it for 20 s. Acquisition training contained four trials a day for five consecutive days. The probe trial is used to test the memory of animals. In the sixth day, the hidden platform was removed and each mouse was allowed to swim freely for 60 s. The swimming pathway, escape latency of mice to find the hidden platform, and the times in the target quadrant were recorded by a digital device connected to a computer.

### Golgi Staining

The mice were anesthetized by chloral hydrate and perfused intracardially with 400 mL normal saline containing 0.5% sodium nitrite, followed by 400 mL 4% formaldehyde solution and the Golgi dye solution containing 5% chloral hydrate, 4% formaldehyde, and 5% potassium dichromate. After being perfused, the brains were dissected into 5-mm × 5-mm sections and transferred to a vial containing Golgi dye solution for 3 days in dark, then immersed in solution containing 1% silver nitrate for another 3 days. The brains were serially sectioned into 40-μm-thick sections using a vibrating microtome (Leica, VT1000S, Germany). The number of spines was counted manually from the two-dimensional projections. Classification of mushroom spines was performed as described previously (Zhao et al., 2006).

### CK2 Activity Assay

CK2 activity in the cell extracts and brain lysates were measured using the CK2 kinase Assay/Inhibitor Screening kit CY-1170 according to the manufacturer’s procedure (Cyclex).

### Primary Hippocampus Neuron Culture

Primary hippocampus neurons were prepared from E17 to E18. Hippocampus were dissected and gently minced in Hank’s buffered saline solution, then suspended in 0.25% (vol/vol) trypsin solution at 37°C for 15 min. Neurons were cultured in neurobasal medium supplemented with 2% (vol/vol) B-27 and 1× GlutaMAX for AAV infection. All cell culture reagents were purchased from Thermo Fisher Scientific.

### Cell Culture and Transfection

HEK293 cells were stably transfected with the longest human tau (tau441) (HEK293/tau). The cells were cultured in Dulbecco’s modified Eagle’s medium (Gibco, Invitrogen; Bleiswijk, Netherlands) in the presence of 200 mg/mL G418 containing 10% fetal bovine serum, and in a humidified incubator aerated with 95% air and 5% CO_2_ at 37°C. HEK293/tau cells were seeded into 6-well plates 1 day before transfection, performed using Lipofectamine 2000 (Invitrogen), according to the manufacturer’s instruction. Forty-eight hours after transfection with SET [wild type (WT)] or mutants respectively, cells were rinsed twice in ice-cold PBS (pH 7.5) and lysed with buffer containing 50 mM Tris-Cl, pH 8.0, 150 mM sodium chloride, 1% NP-40, 0.5% sodium deoxycholate, 0.1% sodium dodecyl sulfate (SDS), 0.02% sodium azide, 100 mg/mL phenylmethysulfonyl fluoride, and 10 mg/mL protease inhibitors (leupeptin, aprotinin, and pepstatin) followed by sonication for 5 s on ice. After centrifugation at 12,000 *g* for 5 min at 4°C, supernatants were fetched out and added with equal volume of 2 Laemmli sample buffer. Samples were boiled for 10 min before electrophoresis. Protein concentration was estimated by BCA kit (Pierce, Rockford, IL, United States).

### Western Blotting

Mice brain tissues, cortex, and hippocampus, were quickly dissected out and homogenized on ice to generate 12% (w/v) homogenate in buffer containing 50 mM Tris⋅HCl pH 8.0, 150 mM NaCl, 1% (vol/vol) Triton X-100, 1 mM EDTA, 1 mM MgCl_2_, 10% (vol/vol) glycerol, 1:100 PMSF, 1:1,000 protease inhibitor mixture containing 4-(2-Aminoethyl)-benzenesulfonyl fluoride hydrochloride, aprotinin, bestatin, leupeptin, E-64, and pepstatin A. The proteins in the extracts were separated by SDS/PAGE and analyzed by Western blotting using antibodies. Samples of HEK293/tau cell extracts were similarly analyzed. Immunoreactive bands were visualized with the Odyssey Infrared Imaging System (LI-COR Biosciences) and quantitatively analyzed by ImageJ software.

### Immunofluorescence

Cultured cells were fixed in 4% (vol/vol) paraformaldehyde for 15 min and permeabilized in phosphate buffer containing 0.5% Triton X-100 (PBST). Non-specific binding was blocked by incubation in PBST buffer containing 0.1% Triton X-100 and 5% (wt/vol) BSA for 1 h. The primary antibodies against pSET (1:100), pSTAT1 (1:500), or CK2 (1:500) were then applied in blocking solution and incubated at 4°C overnight. The secondary antibodies conjugated to Alexa-Fluor 488/548 were added to the coverslip for 1 h at room temperature, and then Hoechst (1:1,000) for 30 min. The coverslips were washed and mounted onto slides. Images were acquired using a laser two-photon confocal microscope (LSM710, Zeiss, Germany).

### LTP

Mice (2 month-old) were used for all our electrophysiology experiments. Mice were deeply anesthetized as mentioned above. When all pedal reflexes were abolished, brains were removed and placed in ice-cold oxygenated slicing solution containing the following: 225 mM sucrose, 3 mM KCl, 1.25 mM NaH_2_PO_4_, 24 mM NaHCO_3_, 6 mM MgSO_4_, 0.5 mM CaCl_2_, and 10 mM D-glucose. Coronal slices (350-μm thick) containing the dorsal hippocampus were cut at 4–5°C in the slicing solution using a Leica VT1000S vibratome and then transferred to an incubation chamber filled with oxygenated slicing solution in a 30°C water bath for 1 h before recording. Slices were laid down in a chamber with an 8 × 8 microelectrode array in the bottom planar (each 50 μm × 50 μm in size, with an interpolar distance of 150 μm) and kept submerged in artificial cerebrospinal fluid (aCSF; 1–2 mL/min) with a platinum ring glued by a nylon silk. Signals were acquired using the MED64 System (Alpha MED Sciences, Panasonic). The fEPSPs in CA1 neurons were recorded by stimulating the Schaeffer fibers from DG. LTP was induced by applying three trains of high-frequency stimulation (HFS; 100 Hz, 1-s duration).

### Hippocampal Stereotactic Injection

Bilateral hippocampus DG zone of mice were injected with AAV respectively as described previously (Zhao et al., 2006). The injection site for adult mice were estimated by using the position of the bregma as reference: anteroposterior, 2 mm; lateral, -1.6 mm; ventral, -2.1 mm.

### Statistical Analyses

Data were expressed as means ± standard deviation (SD) and analyzed using commercial software (GraphPad Prism; GraphPad Software, Inc., La Jolla, CA, United States). The two-way analysis of variance or one-way analysis of variance, or a Student’s *t*-test was used to determine the different means among the groups. The level of significance was set at *P* < 0.05.

## Results

### CK2 Activation Is Accompanied by SET Hyperphosphorylation in an Age-Dependent Manner in AD Mice

**FIGURE 1:**
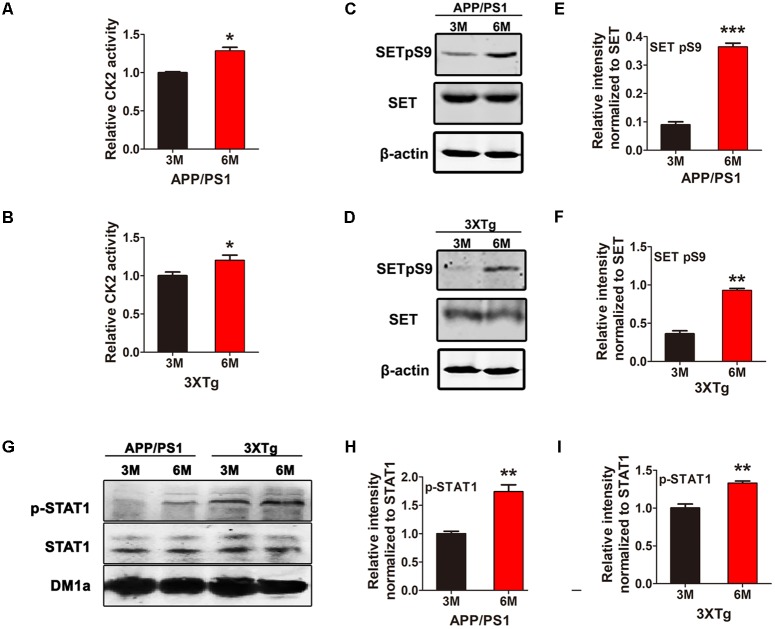
CK2 activation is accompanied by SET hyperphosphorylation in AD mice. CK2 activity in brain of 3 and 6 month old APP/PS1 **(A)** and 3×Tg **(B)** AD mice was measured. **(C–F)** Immunoblotting and quantitative analysis of phosphorylated SET in both AD model, **(G–I)** while STAT1 as a positive control. *n* = 3, all data represent mean ± SEM (*t*-test), ^∗^*P* < 0.05, ^∗∗^*P* < 0.01, ^∗∗∗^*P* < 0.001 vs. 3 month old mouse.

CK2 is activated in AD and we have previously showed CK2 phosphorylates SET in HEK293/tau cells (Yu et al., 2013; Rosenberger et al., 2016), however, whether this event is involved in AD remains unclear. Firstly, we investigated CK2 kinase activity and SET phosphorylation in two AD animal models. We have observed that CK2 kinase activity was markedly elevated in both APP/PS1 (**Figure 1A**) and 3xTg AD (**Figure 1B**) mouse models from 3 to 6 month age. Immunoblotting analysis and quantification support that SET Ser9 again was highly phosphorylated in 6-month-old mice compared with 3-month-old in both animal models (**Figures 1C–F**). In WT C57 mice, no difference was observed in CK2 activity and SET phosphorylation from 3 to 6 month age (**Supplementary Figure S1**). As a substrate of CK2 (Aparicio-Siegmund et al., 2014), the positive control STAT1 was also highly phosphorylated in APP/PS1 and 3×Tg AD older mice (**Figures 1G–I**). These data implicate that CK2 activation in AD may bring along with SET hyperphosphorylation in an age-dependent manner.

### AD-Related CK2 Activation Induces SET Phosphorylation and Cytoplasmic Translocation

**FIGURE 2:**
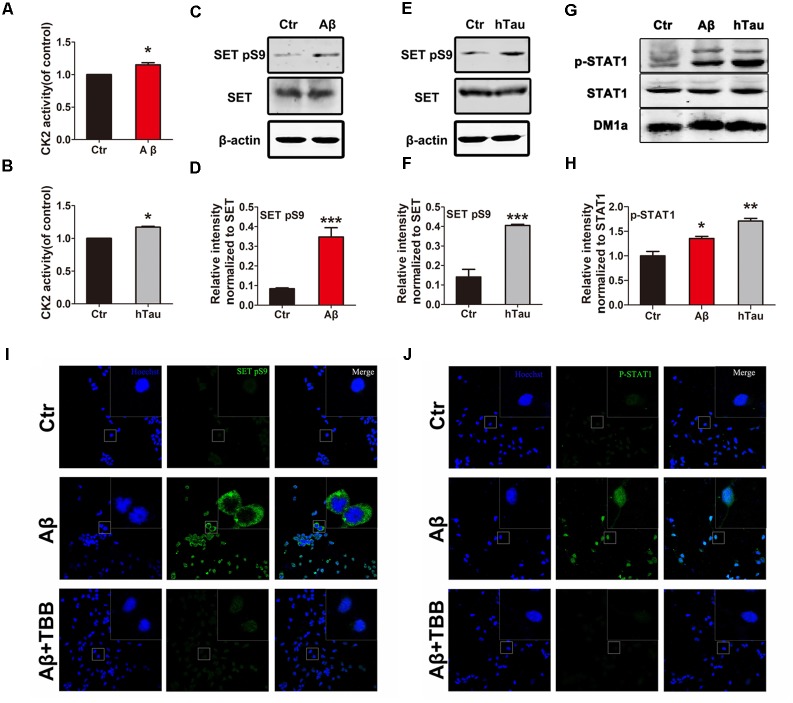
Alzheimer disease (AD)-related CK2 activation induces SET phosphorylation and its cytoplasmic translocation. Primary neuronal culture were treated with Aβ **(A)** or AAV2- hTau **(B)** for 72 h. CK2 activity was detected by the CK2 kinase Assay/Inhibitor Screening kit CY-1170. The lysate from Aβ **(C,G)** or AAV2- hTau **(E,G)** treated primary neuronal culture was collected for Western blots using antibodies against SET, SET pS9 (phospho S9), STAT1, STAT1 pY701, β-actin and DM1A, *n* = 3. **(D,F,H)** Quantitative analysis of the blots in **(C,E,G)** respectively. Immunofluorescence showed subcellular localization and phosphorylation of SET **(I)** and STAT1 **(J)** in primary neuronal culture under treatment with Aβ and TBB, a CK2 inhibitor. Scale bar in **(I,J)**, 150 μm; in insets, 25 μm, *n* = 4. All data represent mean ± SEM (*t*-test), ^∗^*P* < 0.05, ^∗∗^*P* < 0.01, ^∗∗∗^*P* < 0.001 vs. control (Ctr).

To test the possibility that CK2 activation causes SET hyperphosphorylation in AD, we treated primary neuronal culture with Aβ and analyzed CK2 kinase activity. We found that Aβ stimulus resulted in a marked increase in CK2 activity (**Figure 2A**). A similar result was observed by infecting primary neuronal culture with an adeno-associated virus type 2 (AAV2) coding for human Tau (AAV2-hTau) (**Figure 2B**). Immunoblotting validated SET Ser9 was highly phosphorylated following treatment with Aβ (**Figures 2C,D**) and human tau overexpression (**Figures 2E,F** and **Supplementary Figure S2A**). Moreover, treatment with TBB, a CK2 inhibitor, significantly decreased the SET Ser9 hyperphosphorylation induced by Aβ or hTau (**Supplementary Figures S2B–D**). Positive control STAT1 was also highly phosphorylated in both conditions by CK2 (**Figures 2G,H**).

SET is a nuclear protein which translocate to the cytoplasm in AD (Tanimukai et al., 2005; Tsujio et al., 2005). To demonstrate whether Aβ-induced CK2 activation mediates SET subcellular mislocalization, we performed immunofluorescence (IF) staining analysis on primary neuronal culture. We found that Aβ induced p-SET Ser9 cytoplasmic translocation from the nucleus, while inhibition of CK2 by TBB abolished this event (**Figure 2I** and **Supplementary Figure S2E**). As positive control, STAT1 was highly phosphorylated and strongly localized in the nucleus. Treatment with TBB abolished STAT1 phosphorylation (**Figure 2J**). Hence our data suggest that Aβ or tau can induce CK2 activation and may subsequently lead to SET Ser9 phosphorylation, resulting in its cytoplasmic translocation.

### Overexpression of CK2 Induces Cognitive Deficits

**FIGURE 3:**
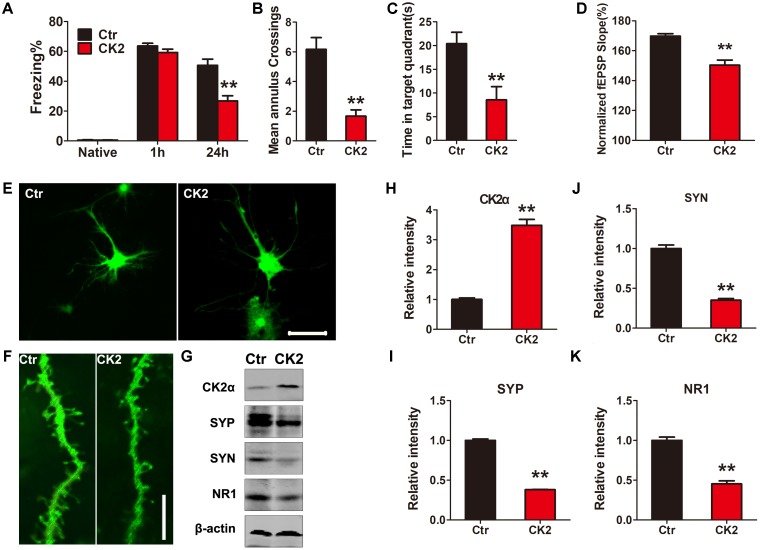
CK2 overexpression induces cognitive deficits. **(A)** After 1 month AAV-CK2 infection, fear conditioning was used to measure the contextual memory. After 1 and 24 h, the mice were put into the same training chamber without shocks, and the total freezing time in 3 min was recorded with a video camera. **(B)** Morris water maze (MWM) test was employed to assess to learning and memory functions in the spatial reference memory task. Compared with the control group, the CK2 mice showed a marked decrease in times of crossing target quadrant and **(C)** the time spent in the target quadrant during the transfer test, *n* = 7. **(D)** Quantitative analyses for fEPSPs relative to baseline after high-frequency stimulation (HFS; 100 Hz, 1-s duration) (*t-*test), *n* = 3. **(E)** The primary hippocampal neurons were infected with control AAV2 or AAV2-CK2 for 48 h, the representative images showed the neuron and **(F)** spine density visualized using two-photon confocal laser scanning microscopy. Scale bars in **(E)**, 50 μm; in **(F)**, 5 μm, *n* = 3. **(G)** The lysate from primary neuronal culture after 48 h viral infection was collected for Western blots using antibodies against CK2α, synaptophysin (SYP), synapsin1 (SYN), NR1, and β-actin. Quantitative analysis of the blots in **(H–K),** respectively (*t*-test), *n* = 3. **(L)** After behavioral tests, immunofluorescence with anti-SET pS9 showed SET intracellular distribution in different sections of cortex and hippocampus including dentate gyrus, CA1, and CA3. Overexpression of CK2 resulted in SET phosphorylation and its cytoplasmic translocation. Scale bar in L, 50 μm; in insets of L, 10 μm, *n* = 3. All data represent mean ± SEM, ^∗∗^*P* < 0.01, vs. control (Ctr).

To demonstrate whether CK2 activation induces any cognitive defects, we injected C57/BL6 mice dentate gyrus with AAV2 coding for CK2 (AAV2-CK2). In 1 month, immunofluorescent analysis showed that SET translocated from nucleus to cytoplasm in hippocampus dentate gyrus zone (**Supplementary Figure S3**), while SET was prominently phosphorylated in CK2 injected mice (**Supplementary Figures S4A,B**, **S5A**). The quantification of cells with cytosolic p-SET in cortex and hippocampus further implicated that SET phosphorylation led to its cytoplasmic translocation (**Supplementary Figure S5B**). Immunoblotting validated that overexpression of CK2 significantly increased SET phosphorylation at Ser9 compared to the control (**Supplementary Figure S6**). Next, we performed fear conditioning experiment. We found that overexpression of CK2 strongly incurred the cognitive deficit (**Figure 3A**). Moreover, MWM test also showed that CK2 overexpression triggered the cognitive impairments (**Figures 3B,C** and **Supplementary Figure S7A**). However, overexpression of CK2 did not affect the motor activity because the swimming speed remained comparable in control and CK2 overexpressed animals (**Supplementary Figure S7B**). To demonstrate indeed CK2-triggered cognitive defect is associated with synaptic plasticity deficit, we performed electrophysiology analysis (**Supplementary Figure S8A**). We found that overexpressed CK2 substantially decreased the fEPSP slope, supporting that CK2 amplification damaged or sabotaged the synaptic plasticity (**Figure 3D** and **Supplementary Figure S8B**). To further investigate whether CK2 overexpression affects the synapses, we infected primary hippocampal neurons with control AAV2 or AAV2-CK2. In 3 days, we conducted immunofluorescence staining, and found that overexpression of CK2 decreased the total dendritic length and the average number of dendritic branches. Noticeably, the spine density and mushroom type were reduced by CK2 overexpression as well (**Figures 3E,F** and **Supplementary Figures S9A–D**). To support this observation, we also performed immuno-blotting analysis of a panel of synaptic proteins in the hippocampus of CK2 mice and in primary hippocampal neurons. We found that numerous synaptic proteins including synaptophysin, synapsin1, and NR1 were evidently reduced after CK2 infection (**Figure 3G** and **Supplementary Figure S10A**), and the quantitative data were summarized in **Figures 3H–K** and **Supplementary Figure S10B**. Thus, our data strongly support that CK2 overexpression and its activation initiates the cognitive defects while impairing synaptic plasticity and synaptogenesis.

### Phosphorylation of SET Induces PP2A Inhibition and Tau Hyperphosphorylation

**FIGURE 4:**
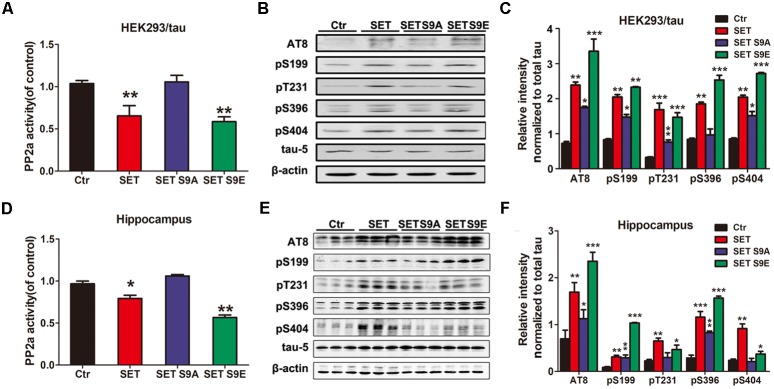
Phosphorylation of SET induces PP2A inhibition and tau hyperphosphorylation. **(A)** HEK293/tau cells were transfected with vector (Ctr), SET, SET S9A, or SET S9E plasmids respectively, and after 48 h transfection the lysates were collected for measurement of PP2A activity and Western blots **(B)** using AT8, pS199, pT231, pS396, pS404, tau5 (total tau), and β-actin. **(C)** Quantitative analysis of the blots showed levels of phosphorylated tau normalized with total tau levels in transfected cells (one-way ANOVA). ^∗^*P* < 0.05, ^∗∗^*P* < 0.01, ^∗∗∗^*P* < 0.001, vs. Ctr (*n* = 3). **(D)** After 1 month infection of AAV2-Vector, AAV2-SET, AAV2-SET S9A, and AAV2-SET S9E in C57/BL6 mice, the hippocampal homogenate were collected for PP2A activity. ^∗^*P* < 0.05, ^∗∗^*P* < 0.01, vs. Ctr. **(E)** Western blots using AT8, pS199, pT231, pS396, pS404, and tau5 showed tau phosphorylation and the level of total tau in hippocampus, and the quantitative analysis was performed **(F)** (one-way ANOVA). All data represent mean ± SEM, ^∗^*P* < 0.05, ^∗∗^*P* < 0.01, ^∗∗∗^*P* < 0.001, vs. control (Ctr), *n* = 3.

Previous studies have showed that CK2 immunoreactivity is markedly increased in NFTs-bearing hippocampal neurons with strong anti-tau immunolabeling, compared with those without NFTs (Masliah et al., 1992; Rosenberger et al., 2016). In the present study, the effect of CK2 on tau phosphorylation was detected *in vitro* and *in vivo*. Immunoblotting showed that tau was highly phosphorylated either in CK2 transfected HEK293/tau cells (**Supplementary Figures S11A,C**) or CK2 infected mice (**Supplementary Figures S11B,D**). To further investigate whether CK2-induced tau hyperphosphorylation is mediated via SET phosphorylation, HEK293/tau cells were transfected with WT SET and different SET mutants including non-phosphorylation SET S9A and phosphorylation mimetic SET S9E (**Supplementary Figures S12A–C**). We found that overexpression of WT SET led to significant reduction in PP2A phosphatase activity, which was further reduced by SET S9E, while non-phosphorylation SET S9A displayed no any inhibitory activity (**Figure 4A**). Next, we monitored tau phosphorylation status with different antibodies in immunoblotting analysis. Tau phosphorylation at Ser199, Ser202/Thr205 (AT8), Thr231, Ser396, and Ser404 sites tightly correlated with PP2A phosphatase activity (**Figure 4B**), and the quantitative data was summarized in **Figure 4C**.

### SET S9E Overexpression Leads to PP2A Inhibition and Cognitive Impairments in C57/BL6 Mice

To assess whether SET phosphorylation is responsible for CK2 overexpression-induced cognitive defects, we injected different AAV2 (WT SET, non-phosphorylation S9A, and phosphorylation mimetic S9E) into the hippocampus of C57/BL6 mice (**Supplementary Figures S12D–F**). In 1 month, we analyzed PP2A enzymatic activity. As expected, overexpression of SET significantly inhibited PP2A activity. Noticeably, phosphorylation mimetic SET S9E displayed much stronger inhibitory activity, by contrast, SET S9A exhibited no inhibitory activity (**Figure 4D**). Immunoblotting from hippocampal (**Figure 4E**) and cortical (**Supplementary Figure S13A**) tissues revealed that the phosphorylation of tau also tightly correlated with the phosphatase activity of PP2A. The quantification data were summarized in **Figure 4F** and **Supplementary Figure S13B**. These data further demonstrate that SET phosphorylation inhibits PP2A activity and subsequently induces tau hyperphosphorylation.

**FIGURE 5:**
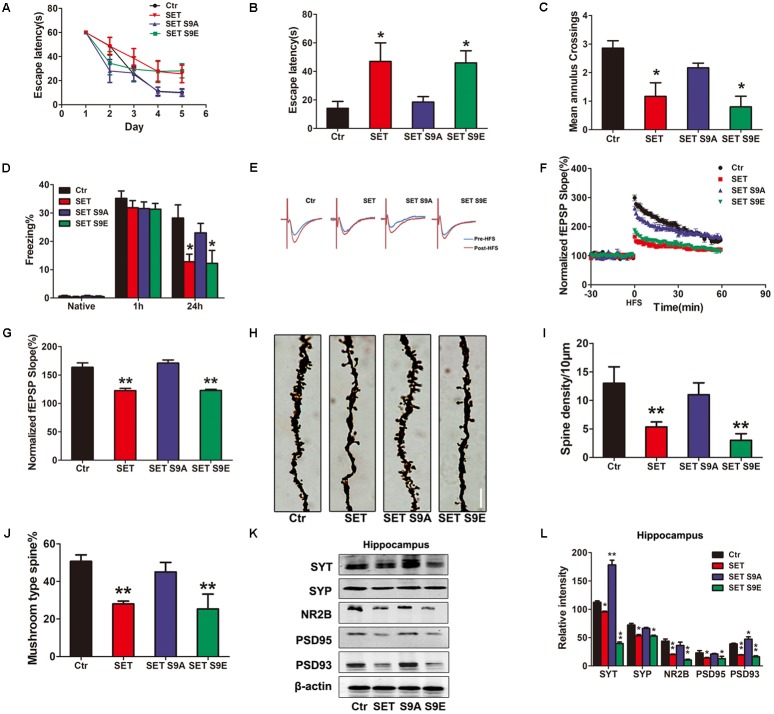
SET S9E mice display cognitive impairments. **(A–C)** MWM test and **(D)** fear conditioning were performed to assess the mice behavior after 1 month viral infection in mice (*n* = 8 per group). **(E)** LTP was induced by applying three trains of HFS (100 Hz, 1-s duration). The traces are average fEPSPs before (blue) and after (red) LTP induction. **(F)** The slope of fEPSP was normalized by the baseline after HFS recorded on hippocampal slices after 1 month infection with AAV2-Vector, AAV2-SET, AAV2-SET S9A, and AAV2-SET S9E in mice. **(G)** Quantitative analyses for fEPSPs relative to baseline after HFS (100 Hz, 1-s duration) (one-way ANOVA), *n* = 3. **(H)** The spine density in hippocampus subset imaged by Golgi staining. **(I,J)** Golgi staining was used to evaluate the dendritic spines density after 1 month viral co-infection in mice (*n* = 3). The quantification for spine density per 10 μm and the ratio of mushroom type in total spine (one-way ANOVA). **(K)** The level of synaptotagmin (SYT), synaptophysin (SYP), NR2B, PSD95, PSD93, and β-actin in the hippocampus was detected 1 month after viral infection in mice, and the quantitative analysis was performed **(L)** (one-way ANOVA), *n* = 3. All data represent mean ± SEM, ^∗^*P* < 0.05, ^∗∗^*P* < 0.01, vs. control (Ctr).

Next, we performed the behavioral assay. We employed MWM and fear conditioning assays. MWM demonstrated overexpression of WT SET or S9E significantly induced the cognitive defects. By contrast, SET S9A overexpressed mice remained comparable with WT control (**Figures 5A–C**). We made similar observations in fear conditioning assay (**Figure 5D**). Electrophysiology also supported that overexpressed WT SET or S9E evidently reduced synaptic plasticity. In contrast, SET S9A displayed no any effect (**Figures 5E–G**). Golgi staining also strongly supported that overexpressed WT SET or S9E significantly reduced spine density and mushroom type, by contrast, SET S9A had no any effect (**Figure 5H**). The quantification was summarized in **Figures 5I,J**. Moreover, we also performed immunoblotting analyses with tissues from hippocampus (**Figure 5K**) or cortex (**Supplementary Figure S14A**) and found that overexpressed WT SET or S9E robustly reduced the synaptic proteins including synaptotagmin, synaptophysin, NR2B, PSD95, and PSD93. Once again, SET S9A had no any effect, indicating that WT SET or its phosphorylation mimetic mutant S9E substantially reduces the synaptic proteins expression, fitting with the Golgi staining observations. The quantified data were summarized in **Figure 5L** and **Supplementary Figure S14B**. Overall, our data strongly suggest that SET phosphorylation induces tau hyperphosphorylation via inhibiting PP2A, leading to the loss of synaptic plasticity.

### Phosphorylation of SET at Ser9 Is Required for CK2-Induced Tau Pathology

**FIGURE 6:**
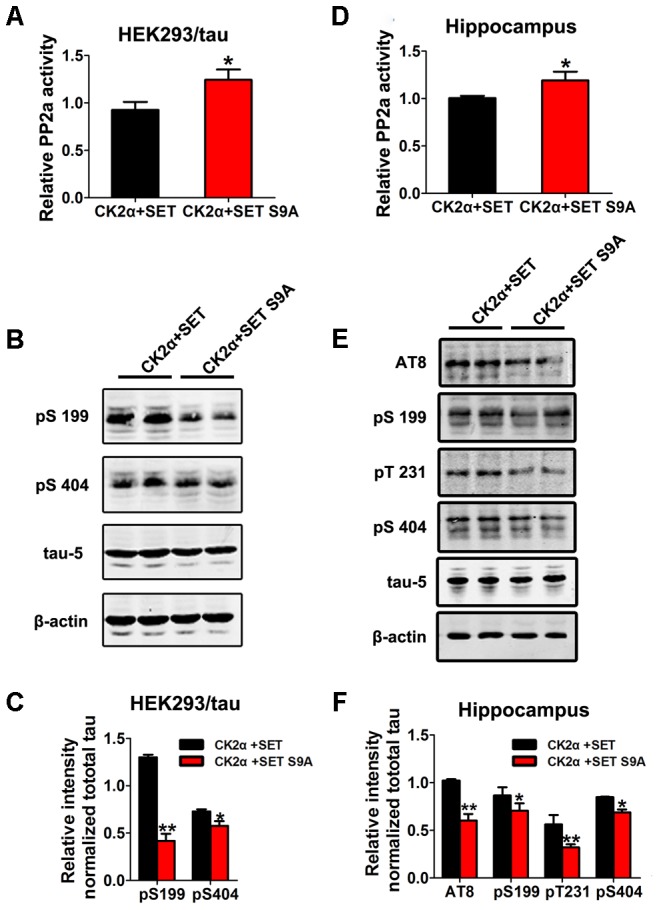
Phosphorylation of SET at Ser9 is responsible for PP2A inhibition and tau hyperphosphorylation in CK2 treated cells and mice. **(A)** HEK293/tau cells were co-transfected with CK2α and SET, CK2α, and SET S9A plasmids for 48 h respectively, and then the cell lysate was collected for measurement of PP2A activity and **(B)** Western blot using pS199, pS404, tau5, and β-actin. **(C)** Quantitative analysis of the blots showed levels of tau phosphorylation normalized with total tau (tau5) levels in transfected cells (*t-*test). **(D)** After 1 month co-infection of AAV2-CK2α + AAV2-SET or AAV2-CK2α + AAV2-SET S9A in C57/BL6 mice, the hippocampal homogenates were collected for PP2A activity. **(E)** Western blots using AT8, pS199, pT231, pS404, and tau5 showed tau phosphorylation and the level of total tau in hippocampus, and the quantitative analysis was performed **(F)** (one-way ANOVA). All data represent mean ± SEM, ^∗^*P* < 0.05, ^∗∗^*P* < 0.01, vs. CK2α+SET, *n* = 4.

To test whether SET phosphorylation by CK2 is really responsible for its overexpression-induced cognitive deficit, we firstly transfected HEK293/tau cells with WT SET or non-phosphorylation SET S9A in the presence of CK2 (**Supplementary Figures S15A–C**). PP2A phosphatase activity assay supported that SET S9A transfected cells displayed much higher phosphatase activity than WT SET (**Figure 6A**). Immunoblotting assay of p-tau tightly correlated with the phosphatase activity (**Figure 6B**). The quantified data were summarized in **Figure 6C**. Together, these data strongly suggest that phosphorylation of SET at Ser9 is responsible for CK2 activation-induced cognitive defect through blocking PP2A phosphatase activity.

To investigate whether SET phosphorylation is indeed responsible for this event, we co-injected AAV2 SET S9A with AAV2 CK2 into the hippocampus of C57/BL6 mice (**Supplementary Figures S15D–F**). In 1 month, we performed phosphatase PP2A activity analysis (**Figure 6D**). As expected, tau phosphorylation tightly correlated with PP2A phosphatase activity in both hippocampus (**Figure 6E**) and cortex (**Supplementary Figure S16A**). The quantification of the western blot results was summarized in **Figure 6F** and **Supplementary Figure S16B**. These data further confirm that phosphorylation of SET at Ser9 is required for CK2-induced tau pathology.

**FIGURE 7:**
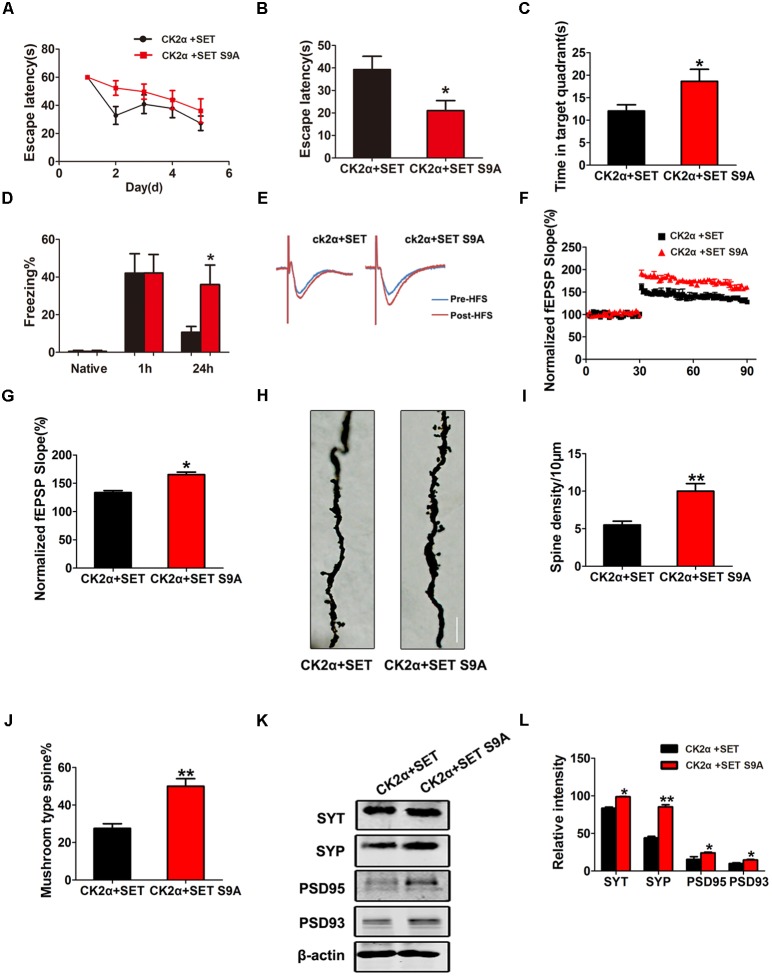
Phosphorylation of SET at Ser9 is required for CK2-induced cognitive defect. **(A–C)** MWM test and **(D)** fear conditioning were performed to assess the mice behavior after 1 month viral infection in C57/BL6 mice, *n* = 8. **(E)** LTP was induced by applying three trains of HFS (100 Hz, 1-s duration). The traces are average fEPSPs before (blue) and after (red) LTP induction. **(F)** The slope of fEPSP was normalized by the baseline after HFS recorded on hippocampal slices after 1 month co-infection with AAV2-CK2α + AAV2-SET or AAV2-CK2α + AAV2-SET S9A in mice. **(G)** Quantitative analyses for fEPSPs relative to baseline after HFS (100 Hz, 1-s duration) (*t*-test), *n* = 3. **(H)** Golgi staining showed SET S9A mice display more dendritic spine and mushroom type spine compared to wild type (WT) SET co-injected mice in the presence of CK2. **(I,J)** Golgi staining was used to evaluate the dendritic spines after 1 month viral co-infection in mice. The quantification for spine density per 10 μm and the ratio of mushroom type in total spine (*t*-test), *n* = 3. **(K)** The level of synaptotagmin (SYT), synaptophysin (SYP), PSD95, PSD93, and β-actin in the hippocampus was detected after 1 month viral co-infection in mice, and the quantitative analysis was performed **(L)** (one-way ANOVA), *n* = 3. All data represent mean ± SEM, ^∗^*P* < 0.05, ^∗∗^*P* < 0.01, vs. CK2α+SET.

Next, we performed MWM and fear conditioning cognitive functional assays. Again, non-phosphorylation SET S9A completely reversed CK2-induced defects in both MWM (**Figures 7A–C**) and fear conditioning assays (**Figure 7D**). Electrophysiology analysis showed that co-injection of non-phosphorylation SET S9A with CK2 significantly increased the fEPSP slope than WT SET co-injection with CK2 (**Figures 7E–G**), supporting non-phosphorylation of SET at Ser9 attenuates the loss of the synaptic plasticity. Notably, Golgi staining indicated that SET S9A animals revealed substantially more dendritic spines and mushroom type spines compared to WT SET co-injected ones in the presence of CK2 (**Figure 7H**), supporting that blocking phosphorylation of SET at Ser9 inhibits the CK2-induced synaptic plasticity impairment and the resulting cognitive defect. The quantified data were summarized in **Figures 7I,J**. Immunoblotting also fitted with this observation as the synaptotagmin, synapsin, PSD95, and PSD93 were clearly increased in SET S9A samples compared with WT SET (**Figures 7K,L**), supporting that the blocking phosphorylation of SET reverses the synaptic defect induced by CK2.

## Discussion

In the current study, we provide extensive evidence demonstrating that CK2 activation by Aβ or tau can induce SET Ser9 phosphorylation, leading to its cytoplasmic translocation, where it blocks PP2A activity, resulting in tau hyperphosphorylation in AD mouse model. Overexpressed phosphorylation mimetic SET S9E mimics the pathology and behavior deficit induced by CK2 strongly supporting that SET Ser9 phosphorylation is responsible for the CK2-mediated pathological effects. On the other hand, we provide additional evidence by co-injecting CK2 virus and non-phosphorylated SET S9A. We found that non-phosphorylated SET reversed CK2 overexpression-induced pathology and cognitive defects. Together, our data strongly support the notion that CK2-mediated phosphorylation of SET induces tau payhology in AD.

SET is a nuclear protein (Tanimukai et al., 2004), which has two major functions. First, SET acts as PP2A binding partner and PP2A inhibitor, leading to tau hyperphosphorylation (Tanimukai et al., 2005). Second, SET can also inhibit DNA nicking in neuronal cell death (Zhang et al., 2014). We have previously reported that Ser9 phosphorylation dictates SET cytoplasmic translocation in AD (Yu et al., 2013). However, it remains unclear how this phosphorylation is molecularly regulated. In the current study, we provide substantial evidence demonstrating that CK2 functions as a pathological trigger of Ser9 phosphorylation on SET and mediates its cytoplasmic translocation in animals. Moreover, we found that SET Ser9 phosphorylation occurs in an age-dependent manner in both APP/PS1 and 3xTg AD mouse models. It has been reported before that SET can be cleaved by the most recently identified protease δ-secretase, which cut SET at N175, leading to its cytoplasmic translocation (Liu et al., 2008; Zhang et al., 2017). It remains unclear how SET phosphorylation and SET cleavage influence its cytoplasmic translocation. On the other hand, it also remains unknown whether SET phosphorylation by CK2 affects its proteolytic cleavage by δ-secretase, or δ-secretase cleavage of SET could mediate its phosphorylation effect by CK2. Further investigation on the crosstalk between these two events is the justification for this study. Moreover, in the current study, we demonstrated that CK2 phosphorylation of SET S9 triggers its cytoplasmic translocation in primary neuronal culture and in animals. Interestingly, it has been reported before that δ-secretase cleavage of SET also induces its cytoplasmic translocation (Liu et al., 2008; Basurtoislas et al., 2013). It is worth noting that both cleaved N-terminal fragment and C-terminal fragment and full-length SET can all bind to PP2A and repress its phosphatase activity (Arnaud et al., 2011). It will be interesting to qualitatively compare whether fragments or phosphorylated SET effect PP2A inhibitory activity. Clearly, to further explore the interaction between SET phosphorylation and its cleavage will provide additional insight into the molecular mechanism of how SET inhibitory activity on PP2A was molecularly regulated. It has been reported before that STAT1 and TDP43 were characterized CK2 substrates (Aparicio-Siegmund et al., 2014; Rudrabhatla, 2014). Here, we further validated STAT1 is highly phosphorylated by CK2 upon Aβ treatment or tau overexpression in primary neuronal culture and two AD mouse models. STAT1 is BACE1 transcription factor (Cho and Jin, 2009). Its phosphorylation by CK2 can induce its nuclear translocation (Aparicio-Siegmund et al., 2014). Presumably, the nuclear translocated STAT1 might up-regulate BACE1 transcription, leading to more Aβ production. Conceivably, in addition to mediating tau pathology, CK2 activity elevation in AD may also affect amyloid beta production by enhancing BACE1 transcription through STAT1 phosphorylation. TDP43 is another major pathological player implicated in ALS (Cykowski et al., 2017). Here, we demonstrate that CK2 is activated in AD and exerts its pathological functions through phosphorylating SET. It is very possible that CK2 might also phosphorylate TDP43 and contribute to ALS neurodegenerative disease. Taken together, our data strongly support that CK2 phosphorylates SET and mediates its translocation by phosphorylating Ser9 on SET. The phosphorylated SET translocates into the cytoplasm, where it binds and represses phosphatase PP2A, leading to tau hyperphosphorylation and NFT pathology. Given that hyperactivation of CK2 in AD exerts numerous deteriorative pathological events, inhibition of CK2 by its inhibitors might provide pharmacological interference for treating AD.

## Conclusion

Our findings show that CK2 phosphorylates Ser9 on SET leading to its cytoplasmic translocation and inhibition of PP2A resulting in tau phosphorylation in AD. This finding provides a novel molecular mechanism for tau pathology in AD development.

## Ethics Statement

All animal experiments were approved by the Animal Care and Use Committee of Huazhong University of Science and Technology and performed in compliance with the National Institutes of Health Guide for the Care and Use of Laboratory Animals.

## Author Contributions

XW designed the research. QZ, YX, YW, YS, KZ, YM, FH, MW, DK, and QW performed the research. BZ, RL, J-ZW, KY, and XW analyzed the data. KY and XW wrote the paper.

## Conflict of Interest Statement

The authors declare that the research was conducted in the absence of any commercial or financial relationships that could be construed as a potential conflict of interest.

## Supplementary Material

FIGURE S1 **(A)** CK2 activity in brain of 3 and 6 month old wild type (WT) mice was measured. CK2 activity was detected by the CK2 kinase Assay/Inhibitor Screening kit CY-1170. **(B)** Immunoblotting and **(C)** quantitative analysis of phosphorylated SET normalized with total SET levels (*t*-test). All data represent mean ± SEM, *n* = 3.

FIGURE S2 Primary neuronal culture was treated with AAV2- hTau **(A)** or Aβ **(B)** and CK2 inhibitor TBB for 72 h. The lysate from treated primary neuronal culture was collected for Western blots using antibodies against SET, SET pS9 (phospho S9), ht7 (human tau antibody), β-actin. **(C,D)** Quantitative analysis of the blots in **(B)** respectively. **(E)** The ratio of cells with cytosolic pSET was quantified in neurons treated Aβ and TBB (one-way ANOVA). All data represent mean ± SEM, ^∗^*P* < 0.05, ^∗∗^*P* < 0.01 vs. control (Ctr), *n* = 3.

FIGURE S3 After 1 month AAV-CK2 infection, immunofluorescence with anti-SET showed SET intracellular distribution in dentate gyrus. Infection of CK2 induced SET cytoplasmic translocation. Scale bar: 50 μm, *n* = 3.

FIGURE S4 **(A)** The lysate from CK2 infected mice was collected for Western blots using antibody against CK2α. **(B)** Quantitative analysis of the blots (*t-*test). All data represent mean ± SEM, ^∗∗^*P* < 0.01 vs. control (Ctr), *n* = 3.

FIGURE S5 **(A)** Immunofluorescence with anti-SET pS9 showed SET intracellular distribution in different sections of cortex and hippocampus including dentate gyrus, CA1, and CA3. Overexpression of CK2 resulted in SET phosphorylation and its cytoplasmic translocation. Scale bar in L, 50 μm; in insets of L, 10 μm, *n* = 3. **(B)** The ratio of cells with cytosolic pSET in different sections of cortex and hippocampus including dentate gyrus, CA1, and CA3 were quantified in CK2 overexpression mice (one-way ANOVA). All data represent mean ± SEM, ^∗^*P* < 0.05, ^∗∗^*P* < 0.01 vs. control (Ctr), *n* = 3.

FIGURE S6 **(A)** The lysate from primary neuronal culture after 72 h viral infection was collected for Western blots using antibodies against SET, SET pS9, and β-actin. **(B)** Quantitative analysis of the blots showing levels of phosphorylated SET normalized with total SET levels in CK2 infected cells (*t*-test). All data represent mean ± SEM, ^∗∗^*P* < 0.01 vs. control (Ctr), *n* = 3.

FIGURE S7 **(A)** After 1 month AAV-CK2 infection, the mice were trained in Morris water maze for six consecutive days to measure the learning capacity. The escape latencies to find the hidden platform were recorded daily. **(B)** The mean swimming speed during the transfer test was no significant difference among each group (*t*-test). All data represent mean ± SEM, *n* = 7.

FIGURE S8 **(A)** LTP was induced by applying three trains of high-frequency stimulation (HFS; 100 Hz, 1-s duration). The traces are average fEPSPs before (blue) and after (red) LTP induction. **(B)** The slope of fEPSP in DG-CA1 was normalized by the base line after HFS, *n* = 3.

FIGURE S9 The primary hippocampal neurons was transfected with AAV2-CK2 or vehicle control (Ctr) for 48 h. The quantification for total dendritic length **(A)**, number of branches **(B)**, the ratio of mushroom type in total spine **(C)** and spine density per 10 μm **(D)**. 30 neurons from three independent cultures were analyzed for each group (*t*-test). All data represent mean ± SEM, ^∗∗^*P* < 0.01 vs. control (Ctr), *n* = 3.

FIGURE S10 **(A)** The lysate from CK2 infected mice was collected for Western blots using antibodies against synaptophysin (SYP), synapsin1 (SYN), and β-actin. **(B)** Quantitative analysis of the blots in **(A)**, respectively (one-way ANOVA). All data represent mean ± SEM, ^∗^*P* < 0.05, ^∗∗^*P* < 0.01 vs. control (Ctr), *n* = 3.

FIGURE S11 The lysate from CK2 transfected HEK293/tau cells **(A)** and CK2 infected mice **(B)** were collected for Western blots using antibody pS199, pS404, Tau5, and β-actin. **(C,D)** Quantitative analysis of the blots (*t*-test). All data represent mean ± SEM, ^∗^*P* < 0.05, ^∗∗^*P* < 0.01, ^∗∗∗^*P* < 0.001 vs. control (Ctr), *n* = 3.

FIGURE S12 **(A)** HEK293/tau cells were transfected with vector (Ctr), SET, SET S9A, or SET S9E plasmids respectively, and after 48 h of transfection the lysates were collected for Western blots using SET pS9, SET, and β-actin. **(B,C)** The quantitative analysis was performed. ^∗^*P* < 0.05 vs. Ctr (*n* = 3). **(D)** After 1 month infection of AAV2-Vector, AAV2-SET, AAV2-SET S9A, and AAV2-SET S9E in C57/BL6 mice, lysates were collected for measurement Western blots using SET pS9, SET, and β-actin. **(E,F)** The quantitative analysis was performed (one-way ANOVA). All data represent mean ± SEM, ^∗^*P* < 0.05 vs. control (Ctr), *n* = 3.

FIGURE S13 **(A)** After 1 month infection of AAV2-Vector, AAV2-SET, AAV2-SET S9A, and AAV2-SET S9E in C57/BL6 mice, the cortex homogenate were collected for Western blots using AT8, pS199, pT231, pS396, pS404, Tau5, and β-actin. **(B)** The quantitative analysis was performed (one-way ANOVA). All data represent mean ± SEM, ^∗^*P* < 0.05, ^∗∗^*P* < 0.01, ^∗∗∗^*P* < 0.001 vs. control (Ctr), *n* = 3.

FIGURE S14 **(A)** The level of synaptotagmin (SYT), synaptophysin (SYP), NR2B, PSD95, PSD93, and β-actin in the cortex was detected after 1 month viral (WT SET, non-phosphorylate S9A, and phosphorylation mimetic S9E) infection in C57/BL6 mice and **(B)** the quantitative analysis was performed (one-way ANOVA). All data represent mean ± SEM, ^∗^*P* < 0.05, ^∗∗^*P* < 0.01 vs. control (Ctr), *n* = 3.

FIGURE S15 **(A)** HEK293/tau cells were co-transfected with CK2α (catalytic alpha subunit) and SET, CK2α and SET S9A plasmids for 48 h respectively, and then the cell lysate was collected for Western blot using SET pS9, SET, and β-actin. **(B,C)** Quantitative analysis was performed. **(D)** After 1 month co-infection of AAV2-CK2α + AAV2-SET or AAV2-CK2α + AAV2-SET S9A in C57/BL6 mice, the homogenate were collected for Western blots using SET pS9, SET and β-actin, and **(E,F)** the quantitative analysis was performed (*t*-test). All data represent mean ± SEM, ^∗^*P* < 0.05 vs. CK2α+SET, *n* = 4.

FIGURE S16 **(A)** After 1 month co-infection of AAV2-CK2α + AAV2-SET and AAV2- CK2α + AAV2-SET S9A in C57/BL6mice, the cortex homogenate were collected for Western blots using AT8, pS199, pT231, pS404, and Tau5 showing tau phosphorylation and the level of total tau, and **(B)** the quantitative analysis was performed (one-way ANOVA). All data represent mean ± SEM, ^∗^*P* < 0.05, ^∗∗^*P* < 0.01 vs. CK2α+SET, *n* = 4.

TABLE S1 Antibodies employed in this study.
