# Supplementary material for: CK2 Phosphorylating I2PP2A/SET Mediates Tau Pathology and Cognitive Impairment
Source: Front Mol Neurosci. 2018 Apr 30;11:146. doi: 10.3389/fnmol.2018.00146 (PMC5936753; doi:10.3389/fnmol.2018.00146)

Supplementary Figure 1

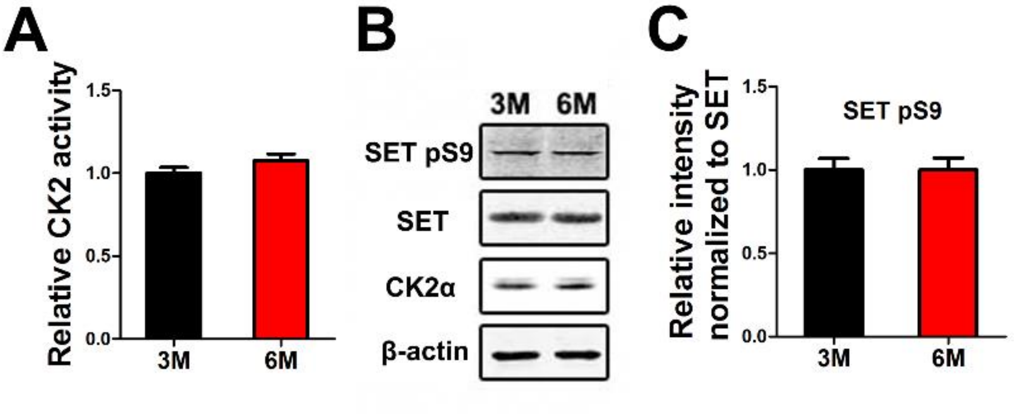

Supplementary Figure 2

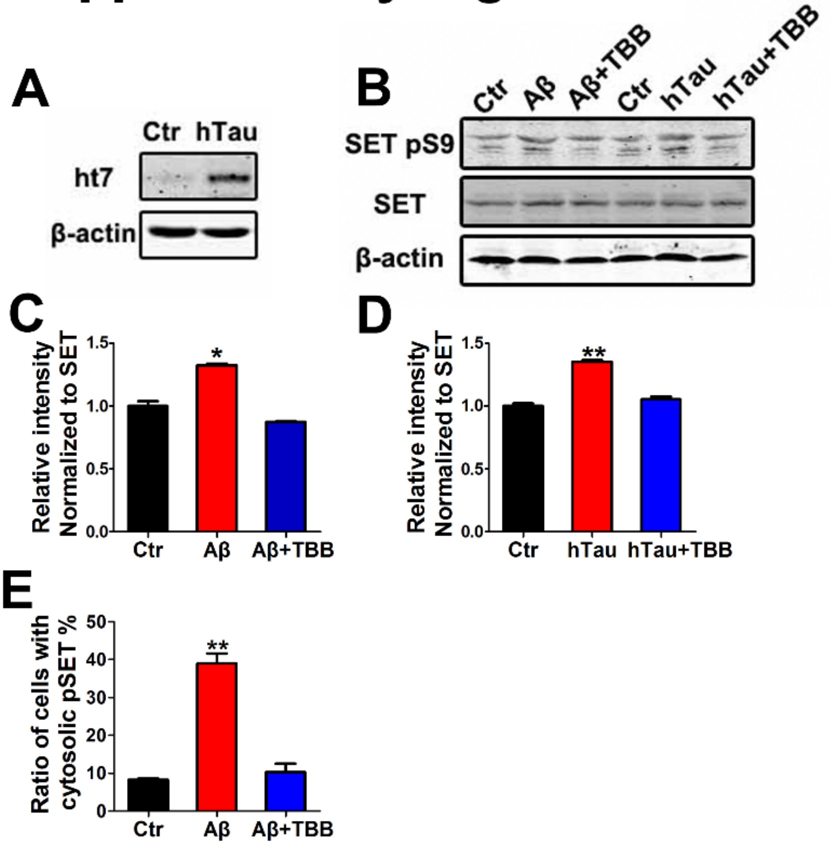

Supplementary Figure 3

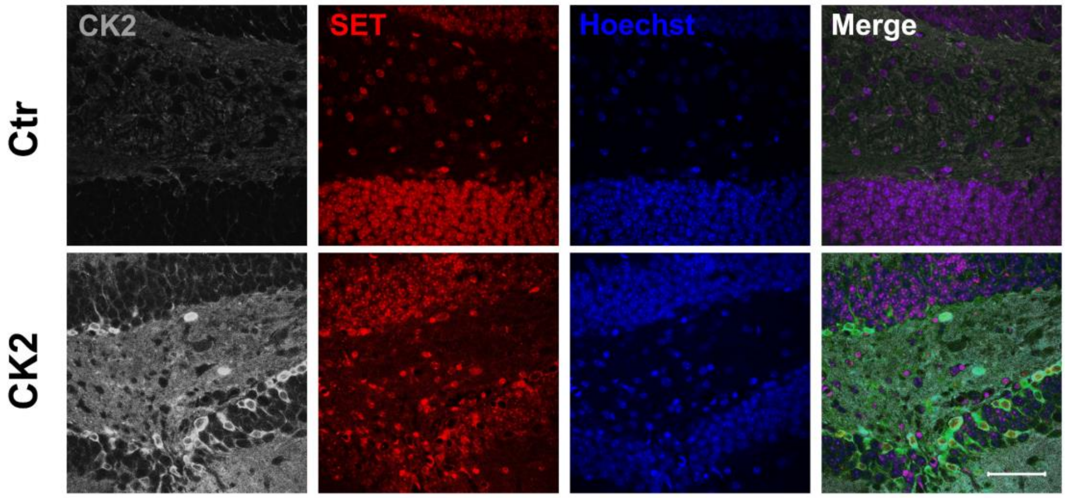

Supplementary Figure 4

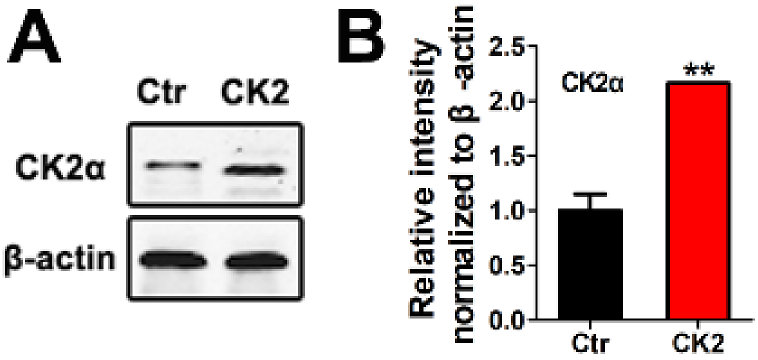

Supplementary Figure 5

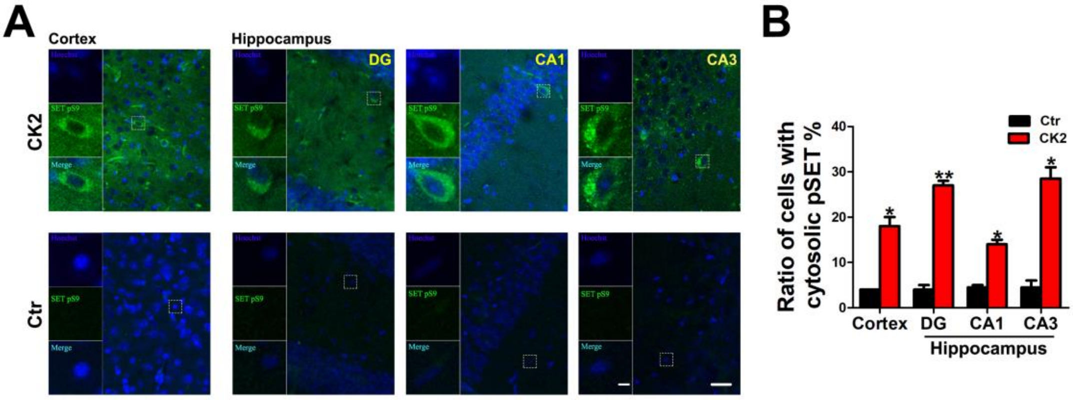

Supplementary Figure 6

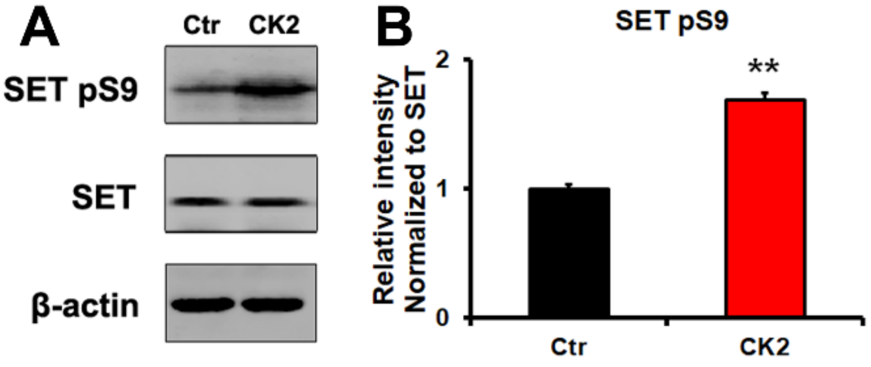

Supplementary Figure 7

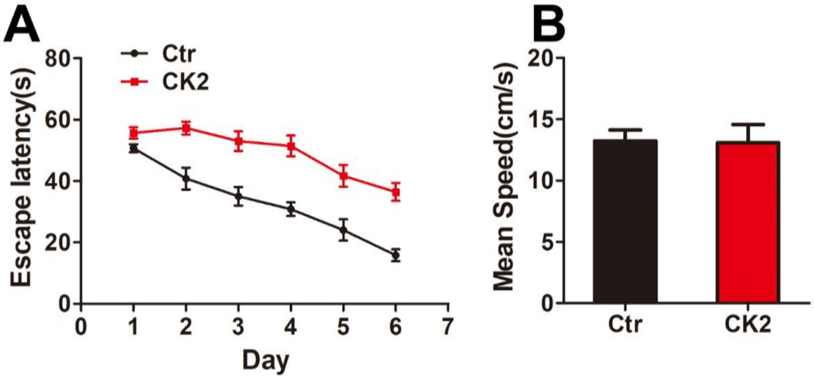

Supplementary Figure 8

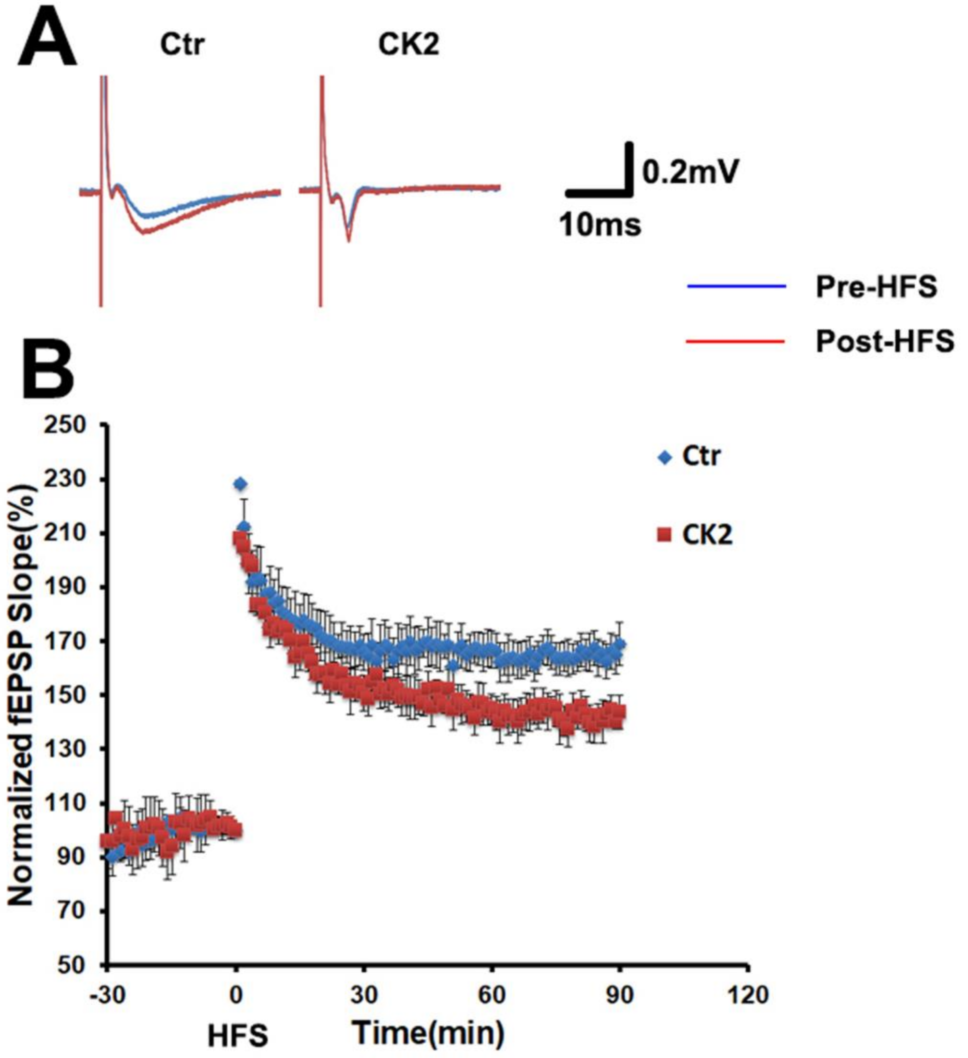

Supplementary Figure 9

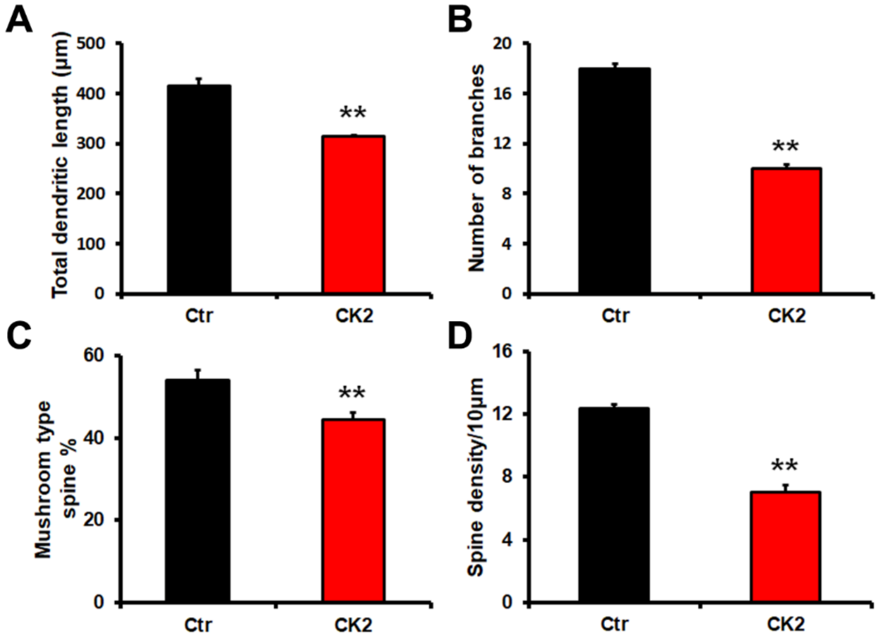

Supplementary Figure 10

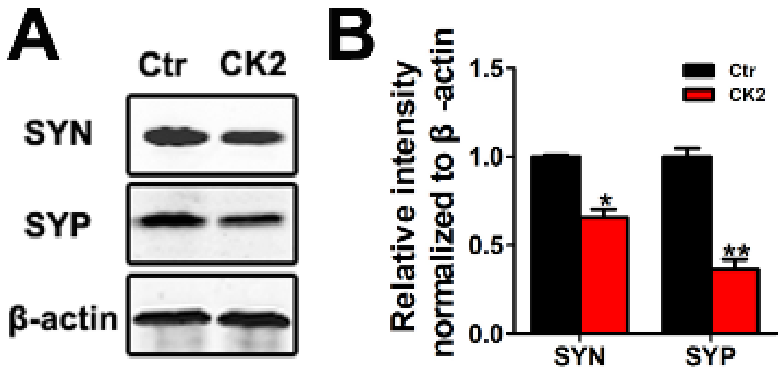

Supplementary Figure 11

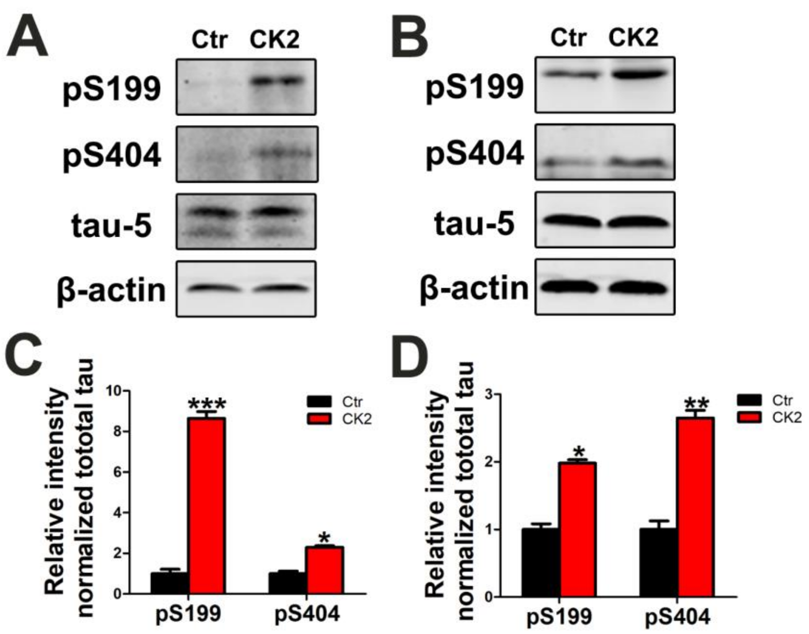

Supplementary Figure 12

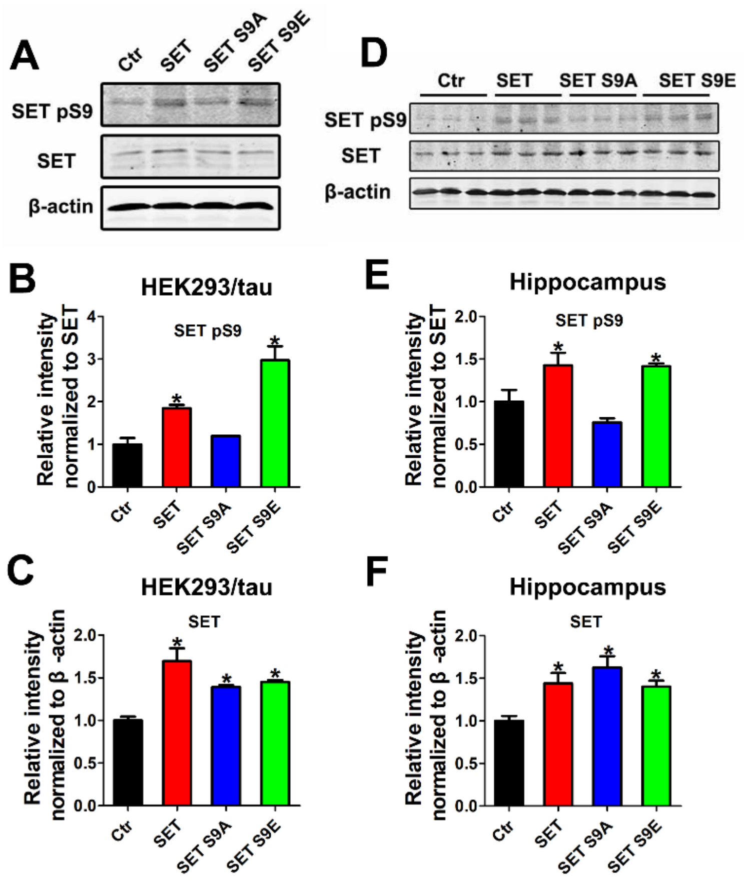

Supplementary Figure 13

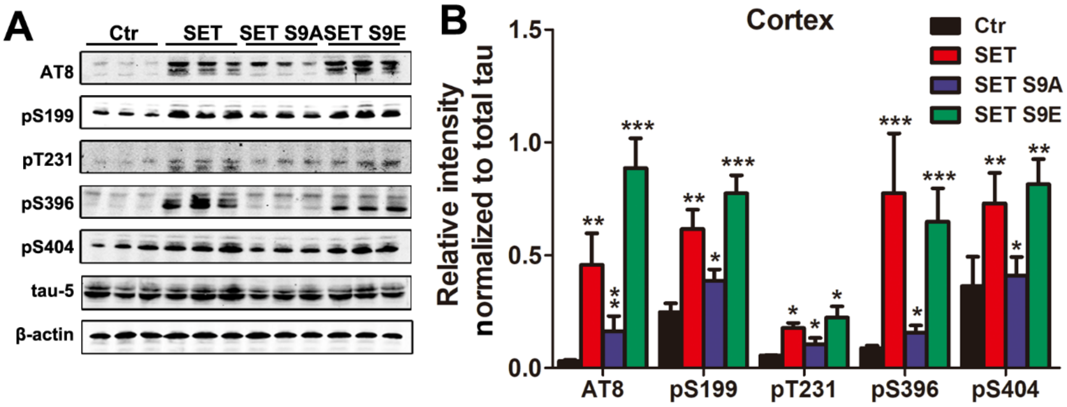

Supplementary Figure 14

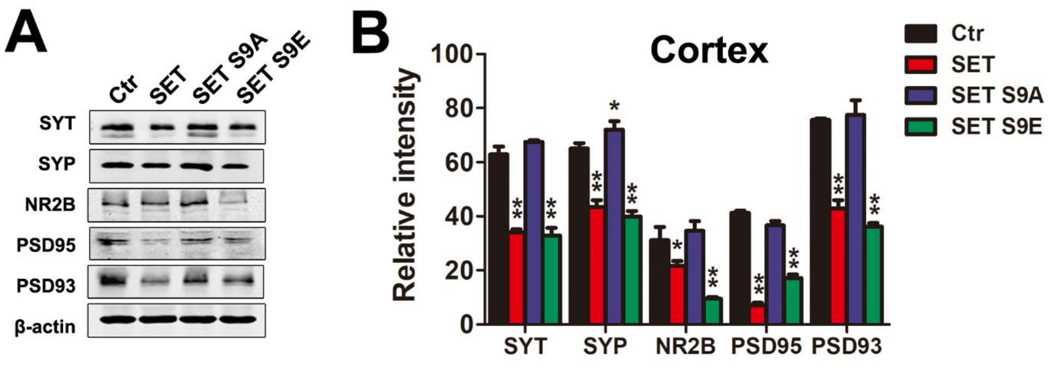

Supplementary Figure 15

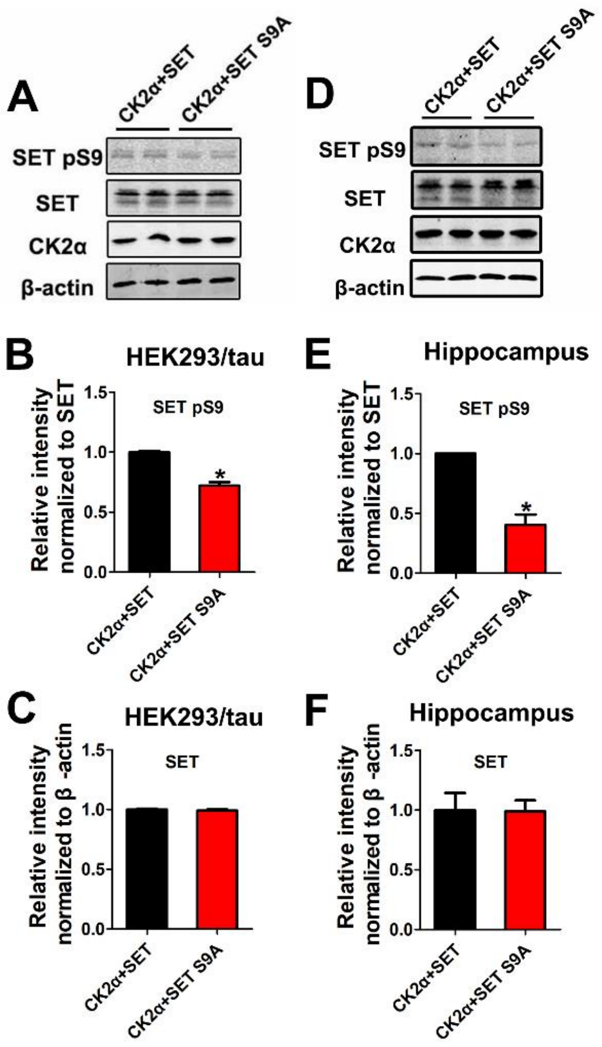

Supplementary Figure 16

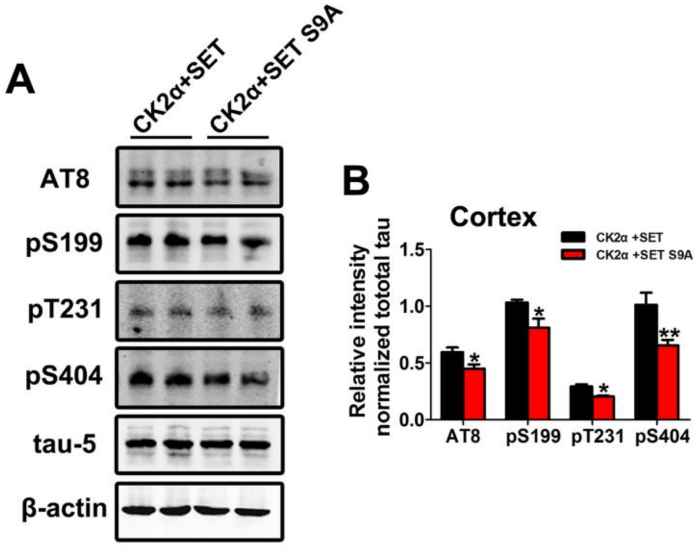

Supplement: FIGURE S1 — (A) CK2 activity in brain of 3 and 6 month old wild type (WT) mice was measured. CK2 activity was detected by the CK2 kinase Assay/Inhibitor Screening kit CY-1170. (B) Immunoblotting and (C) quantitative analysis of phosphorylated SET normalized with total SET levels (t-test). All data represent mean ± SEM, n = 3. [file Image_1.PDF]
