# Supplementary material for: CK2 Phosphorylating I2PP2A/SET Mediates Tau Pathology and Cognitive Impairment
Source: Front Mol Neurosci. 2018 Apr 30;11:146. doi: 10.3389/fnmol.2018.00146 (PMC5936753; doi:10.3389/fnmol.2018.00146)
Supplement: TABLE S1 — Antibodies employed in this study. [file Table_1.DOCX]

**Supplementary Table 1. Antibodies employed in this study**

| **Antibody** | **Clone name/ catalog number** | **Specific** | **Type** | **Dilution** | **Source** |
| --- | --- | --- | --- | --- | --- |
| Anti-SET (phospho S9) | TAF-1 | Phosphorylated SET  at Ser 9 | pAb | 1:200 for WB  1:50 for IF | Abmart |
| Anti-CK2a antibody | 8E5 | CK2a | mAb | 1:1000 for WB  1:100 for IF | Abcam |
| Tau-1 | TAU-1 | Dephosphorylated tau  at Ser198, 199/Ser202 | mAb | 1:25000 for WB | Chemicon |
| Tau-5 | TAU-5 | Total tau | mAb | 1:1000 for WB | Lab Vision |
| Anti-Tau (pS396) | Cat # 710298 | Phosphorylated tau  at Ser396 | pAb | 1:1000 for WB | Biosource |
| Anti-Tau (pS404) | Cat # 44-758G | Phosphorylated tau  at Ser404 | pAb | 1:1000 for WB | Biosource |
| Anti-Tau (pS199) | Cat # 44-734G | Phosphorylated tau  at Ser199 | pAb | 1:1000 for WB | Biosource |
| Anti-Tau (pT231) | Cat # 710126 | Phosphorylated tau  at Thr231 | pAb | 1:1000 for WB | Biosource |
| Anti-Tau (pS262) | **C**at # 44-750G | Phosphorylated tau  at Ser262 | pAb | 1:1000 for WB | Biosource |
| Anti-Tau(pS202,pT205) | AT8 | Phosphorylated PHF tau  at Ser202+ Ser205 | mAb | 1:1000 for WB | Thermo |
| Synaptotagmin | ASV30 | Total synaptotagmin | mAb | 1:1000 for WB | Abcam |
| Synaptophysin | [ab32594](http://www.abcam.cn/synaptophysin-antibody-ab32594.html) | SYP245-258 | pAb | 1:1000 for WB | Abcam |
| β-actin | AC-15 | β-actin | mAb | 1:1000 for WB | Sigma |
| PSD93 | ab2930 | Post Synaptic Density 93 | pAb | 1:1000 for WB | Abcam |
| PSD95 | 2507 | Post Synaptic Density 95 | pAb | 1:1000 for WB | Cell Signaling Technology |
| STAT1 | ab31369 | Total STAT1 | pAb | 1:1000 for WB | Abcam |
| STAT1 pY701 | M135 | Phosphorylated STAT1  at Tyr701 | mAb | 1:1000 for WB | Abcam |
| NR2B | Ab65783 | NMDAR2B C-term | pAb | 1:1000 for WB | Abcam |

CK2, casein kinase 2; IF, immunofluorescence; mAb, monoclonal antibody; pAb, polyclonal antibody; WB, Western blot.
